# Metabolomic insights into the mechanisms of chickpea milkvetch (*Astragalus Cicer* L.) response to salt stress

**DOI:** 10.1186/s12870-026-08375-3

**Published:** 2026-02-26

**Authors:** Dongqiang Wu, Ting Ma, Dongqin Li

**Affiliations:** 1 Gansu Yasheng Tianyuanmuge Pratacultural Industry Group Co., Ltd., Lanzhou, China; 2 Gansu Agricultural University, Lanzhou, China; 3 Gansu Grassland Technical Extension Station, Lanzhou, China

**Keywords:** *A. cicer*, NaCl stress, Roots, Metabolome

## Abstract

**Background:**

Soil salinization severely impacts plant growth and development, limiting the advancement of agro-ecological economies. *Astragalus cicer*, a highly efficient ecological grass, green manure crop, and high-quality protein forage. However, the molecular mechanisms underlying its root response to NaCl stress remain poorly understood. In this study, the *A. cicer* cultivar ‘Ganlü No. 2’ was treated with 150 mM NaCl over different time periods to investigate the physiological and metabolomic mechanisms underlying its adaptation to saline conditions.

**Results:**

*A. cicer* exhibited concentration-dependent growth responses to NaCl treatment: a low salt concentration (50 mM) promoted early growth, whereas a high salt concentration (150 mM) induced pronounced oxidative stress and inhibited plant growth. Metabolomic analysis identified 1,677 differentially accumulated metabolites (DAMs). During the early stress phase, roots rapidly accumulated key signaling molecules, including cAMP, cGMP, and the cytokinin dihydrozeatin, suggesting the swift activation of the cAMP/cGMP signaling pathway to initiate the stress response. By the mid-term phase, the metabolite profile exhibited dynamic adjustments, with sustained high levels of cAMP and dihydrozeatin, while antioxidant metabolites such as rosmarinic acid were significantly downregulated. KEGG pathway enrichment analysis consistently highlighted the enrichment of purine metabolism and cysteine/methionine metabolism across all time points, underscoring the pivotal roles of nucleotide and sulfur-containing amino acid metabolism in the long-term adaptation of roots to NaCl stress. Clustering and Short Time-series Expression Miner (STEM) analysis further revealed significant temporal specificity in DAM expression, with prominent enrichment in pathways such as secondary metabolite biosynthesis, flavonoid biosynthesis, phenylpropanoid metabolism, purine/pyrimidine metabolism, and amino acid biosynthesis.

**Conclusion:**

*A. cicer* exhibits a dynamic adaptive response to NaCl stress, rapidly activating signaling pathways and coordinating the regulation of core metabolic pathways, including nucleotide metabolism, sulfur-containing amino acid metabolism, and secondary metabolism. These findings provide important molecular insights into the salt tolerance mechanisms of this species and offer valuable information for salt-tolerant breeding strategies.

**Supplementary Information:**

The online version contains supplementary material available at 10.1186/s12870-026-08375-3.

## Introduction

Soil salinization is not only a global environmental issue but also a major factor affecting food security [1]. It is well established that high-salinity environments adversely affect plant growth and development, primarily through ion toxicity, oxidative stress, and osmotic stress [2]. Specifically, ion toxicity and the resulting osmotic stress disrupt cellular homeostasis, while reactive oxygen species (ROS) such as hydrogen peroxide and hydroxyl radicals induce lipid peroxidation of cell membranes [3]. Nevertheless, plants can enhance their tolerance to salt stress through physiological and biochemical responses, including mitigating ion and ROS accumulation via stress-adaptive mechanisms or metabolic adjustments [4]. Studies have indicated that stress-tolerant plants have evolved a suite of strategies to cope with abiotic stresses, which can also be leveraged for the restoration of saline soils [5].

Roots are the primary organs for transporting water, nutrients, and stress signals to the shoot and are central to plant adaptation to adverse environments [6]. Salt stress induces lignification and Casparian strip formation in root cells [7], and strengthens the exodermis and endodermis, thereby limiting apoplastic transport from the cortex to the stele [8]. For example, salt-tolerant barley (*Hordeum vulgare*) roots undergo lignification under salt stress, facilitating symplastic water and solute transport while selectively excluding Na^+^ to maintain root growth [9]. In wild soybean (*Glycine soja*), key salt-tolerance strategies include root accumulation of toxic ions with reduced shoot translocation, and enhanced antioxidant enzyme and secondary metabolite production to scavenge ROS [10].

Plants exhibit complex and diverse metabolic pathways throughout growth and development [11]. Metabolomics allows characterization of these metabolic changes, providing insights into plant-environment interactions and mechanisms of ion uptake [12]. Metabolites can also function as signaling molecules, activating protective mechanisms in salt-tolerant plants, including ROS homeostasis, regulation of ion transporters, and phytohormone signaling. Previous studies have used metabolomics to investigate plant responses to salt stress. For instance, high salinity alters metabolite profiles in the halophyte *Nitraria sibirica* [13], and temporal metabolomic analyses in *Arabidopsis thaliana* revealed dynamic cellular metabolic changes under salt stress [14]. Short-term salt stress in *Oryza sativa* induces stress-responsive metabolites through stress-related gene regulation [15], and overexpression of trehalose-6-phosphate phosphatase in transgenic rice enhances trehalose accumulation, increasing chlorophyll content and reducing membrane oxidative damage [16]. In *Arabidopsis* roots, the oxalate pathway contributes to salt adaptation by accumulating both primary and secondary metabolites, including lignin, accompanied by cell wall thickening [17].

*A. cicer* L. (chickpea milkvetch) is an excellent species for soil and water conservation, a high-efficiency green manure crop, and a high-quality protein forage, demonstrating strong adaptability to various environments [18, 19]. It can be cultivated in saline soils, but high salinity significantly inhibits its growth, particularly affecting root development [20]. Therefore, developing salt-tolerant varieties is crucial to enhance its growth and expand its adaptability. However, the physiological and metabolic mechanisms underlying root responses of *A. cicer* to salt stress remain largely unexplored. This study aims to investigate the adaptive mechanisms of root responses to NaCl stress by analyzing physiological changes, reactive oxygen species (ROS) levels, and root metabolite changes under different salt concentrations. Through metabolomic analysis of root metabolites and a comprehensive assessment of physiological indicators (such as antioxidant enzyme activity and compatible solute content), this research will provide insights into the mechanisms of salt stress tolerance. The findings will contribute to a deeper understanding of the salt stress response in *A. cicer* and support the development of salt-tolerant varieties, thereby promoting its widespread application in saline soils.

## Materials and methods

### Plant material and experiment treatment

The experimental material used was the chickpea milkvetch variety *A. cicer* ‘Ganlv 2’, provided by Gansu Chuanglv Grass Technology Co., Ltd. Seeds with uniform size, full grains, and no insect damage were selected, soaked in warm water for 12 h to break dormancy, and subsequently sown in nutrient soil. Seedlings were grown in a tissue culture room under controlled conditions with a 16 h/8 h light/dark photoperiod, temperature of 25 °C during light and 20 °C during dark periods, relative humidity of 60–70%, and light intensity of 5000 lx. When seedlings reached the two-leaf stage, they were transplanted into 96-well hydroponic boxes (12 cm × 8 cm × 11 cm), with twelve seedlings per box, containing 1000 mL of Hoagland nutrient solution. The hydroponic boxes were maintained under the same culture conditions, and the nutrient solution was replaced every three days. At the six-leaf stage, seedlings were subjected to 150 mM NaCl stress. Root samples were collected at 0, 12, 48, and 72 h after treatment. For each time point, roots were sampled from nine hydroponic boxes containing seedlings of uniform growth, totaling 108 plants. Samples were used for physiological measurements, with three biological replicates per indicator. The remaining samples, ensuring six biological replicates per time point, were stored at − 80 °C for subsequent metabolomic analysis.

### Germination experiment

Surface-sterilized seeds were placed in Petri dishes (9 cm in diameter) lined with two layers of filter paper, with fifty seeds evenly spaced per dish. Four milliliters of NaCl solution (0、50、100、150 mM) at different concentrations were added to maintain germination moisture, while distilled water was used as the control. Three biological replicates were established for each treatment. Petri dishes were incubated in a growth chamber under controlled conditions of 25 °C/16°C (light/dark) with a 16 h/8 h photoperiod. Seeds were considered germinated when the radicle became visible, and germination was monitored for 14 days. Ten seedlings were randomly selected from each treatment to measure root and plumule lengths using a ruler, and five uniformly growing seedlings from each dish were used to determine seedling fresh weight, with five seedlings representing one replicate. Germination rate was expressed as the percentage of seeds that germinated within 14 days relative to the total number of seeds sown, while germination viability was calculated as the percentage of seeds that germinated within seven days relative to the total number of seeds. The germination index was calculated by summing, for each day, the ratio of the number of seeds germinated on that day to the corresponding day of germination. Relative salt injury rate was expressed as the percentage reduction in seed germination under salt stress compared with the control. The root-to-plumule ratio was calculated as the ratio of root length to plumule length, and the vigor index was determined by multiplying the germination index by the mean fresh weight of five seedlings [21].

### Determination of H₂O₂, O₂⁻· production, antioxidant enzyme activities, soluble protein, soluble sugar, and malondialdehyde

Hydrogen peroxide (H₂O₂) content in roots was determined using the potassium iodide (KI) colorimetric method. Briefly, fresh root tissue (0.1 g) was homogenized in 0.1% trichloroacetic acid under ice-cold conditions and centrifuged at 4 °C. The supernatant was reacted with phosphate buffer and KI solution, incubated at 28 °C, and absorbance was measured at 390 nm [22].

For enzyme assays, crude enzyme extracts were prepared by homogenizing root tissue (0.5 g) in phosphate buffer (pH 7.8) followed by centrifugation at 4 °C. The supernatant was used to determine superoxide anion (O₂⁻·) production rate, antioxidant enzyme activities, and soluble protein content. The O₂⁻· production rate was quantified using the hydroxylamine method, with absorbance recorded at 530 nm [23].

Superoxide dismutase (SOD) activity was assayed based on its inhibition of nitroblue tetrazolium (NBT) photoreduction and measured at 560 nm. Peroxidase (POD) activity was determined using the guaiacol oxidation method by monitoring absorbance changes at 470 nm. Catalase (CAT) activity was assessed by following the decomposition of H₂O₂ at 240 nm, while ascorbate peroxidase (APX) activity was measured by monitoring the oxidation of ascorbate at 290 nm [24].

Soluble protein content was determined using the Coomassie Brilliant Blue method, with absorbance measured at 595 nm [25].

### Metabolomic analysis

Non-targeted metabolomic analysis was performed on six biological replicates per time point to investigate root metabolic changes under salt stress. Approximately 100 mg of root tissue was ground in liquid nitrogen and transferred to an Eppendorf tube. Subsequently, 0.5 mL of 80% mass spectrometry-grade methanol was added, and the mixture was vortexed and incubated on ice for 5 min. After centrifugation at 5,000 × g for 20 min at 4 °C, the supernatant was collected and diluted with mass spectrometry-grade water to achieve a final methanol concentration of 53%. The mixture was then centrifuged at 15,000 × g for 20 min at 4 °C, and the resulting supernatant was transferred to an autosampler vial. Equal volumes from all experimental samples were pooled to generate a quality control (QC) sample, which was analyzed at the beginning, middle, and end of the injection sequence to monitor analytical stability. Chromatographic separation was performed on a Hypersil Gold column (Thermo Fisher, USA) at 40 °C with a flow rate of 0.2 mL·min⁻¹. The mobile phases consisted of 0.1% formic acid (A) and methanol (B) for both positive and negative ion modes. Mass spectrometric detection was conducted over an m/z range of 100–1500 using an ESI source with a spray voltage of 3.5 kV and an ion transfer tube temperature of 320 °C. Data acquisition alternated between positive and negative polarity using data-dependent MS/MS scans.

Raw LC-MS/MS data were processed using Compound Discoverer 3.3. Metabolite identification was performed using NovoMetDB-UM-V1.0 (a self-built standard and secondary spectral library). Level 1 identification was based on authentic standards, MS1, MS2, and retention time (RT) matching. Database matching required MS2 spectral similarity ≥ 70%, which served as the scoring threshold. Metabolite abundances were normalized to the total ion current (TIC) of each sample. Metabolite identification and classification were conducted using MassList, mzCloud, and mzVault databases. QC performance was evaluated via correlation analysis and principal component analysis (PCA) of all samples. Multivariate statistical analyses, including PCA and partial least squares discriminant analysis (PLS-DA), were applied to identify differential metabolites among comparison groups. Hierarchical cluster analysis (HCA) was used to visualize relationships between samples and metabolites. Differentially accumulated metabolites (DAMs) were defined based on the following criteria: VIP > 1.0 from PLS-DA, fold change (FC) > 1.5 or FC < 0.667, and *P* < 0.05 (Duncan’s test). All downstream analyses, including KEGG pathway annotation, correlation with physiological data, and further functional interpretation, were performed using these selected DAMs.

### Statistical analysis

Statistical analysis was performed using SPSS 20.0 statistical software (SPSS Inc., Chicago, IL, USA). Three biological replicates were used for physiological experiments, and six biological replicates per time point were used for metabolomic analysis. Values were means ± standard error (SE). Multiple comparisons were processed by application of Duncan’s multiple range test to determine the significance of the results among different treatments at a *p* < 0.05 level.

## Result

### Germination and seedling growth of *A. cicer *under NaCl stress

Under NaCl stress, both the germination percentage and vigor index of *A. cicer* seeds initially increased and then declined with rising salinity, reaching their highest levels at 50 mM NaCl. At 150 mM, both parameters were significantly lower than those of the control (CK) (*p* < 0.05), indicating strong inhibition of seed germination (Table 1). Germination energy and the germination index gradually decreased with increasing NaCl concentration, with a pronounced reduction observed from 100 mM onward. The relative salt injury rate exhibited a biphasic response, decreasing at low salinity but increasing markedly under high NaCl stress; notably, 50 mM NaCl promoted seed germination, whereas severe salt injury occurred at 150 mM (Table 1). Regarding seedling growth, shoot length was significantly greater than that of CK at 50 mM NaCl, but was significantly reduced at all higher NaCl concentrations (*p* < 0.05) (Table 2).

**Table 1:** Seed germination of *A. cicer* under NaCl stress

NaCl Concentration (mM)	Germination rate (%)	Germination viability (%)	Germination index	Vigor index	Relative salt rate (%)
0	71.00 ± 4.12a	62.00 ± 3.37a	43.38 ± 2.49a	1.87 ± 0.13a	0.00 ± 2.93c
50	79.00 ± 2.08b	33.00 ± 1.91b	29.51 ± 2.60b	1.94 ± 0.23a	−11.27 ± 2.93d
100	46.00 ± 3.46c	10.00 ± 1.41c	10.59 ± 1.44c	0.33 ± 0.05b	35.21 ± 4.88b
150	9.00 ± 0.58d	2.00 ± 0.00d	2.71 ± 0.42d	0.41 ± 0.01b	87.33 ± 0.81a

**Table 2:** Seedling growth of *A. cicer* under NaCl stress

NaCl Concentration (mM)	The length of root (mm)	The length of plumule (mm)	Root-plumule ratio	The weight of seedling (mg)
0	50.68 ± 3.15a	5.60 ± 0.89b	9.87 ± 1.39a	215.55 ± 3.55b
50	35.56 ± 1.44b	7.18 ± 0.30a	4.96 ± 0.12b	326.85 ± 14.71a
100	5.05 ± 1.46c	5.33 ± 0.28b	0.93 ± 0.31c	157.48 ± 15.27c
150	1.37 ± 0.24 cd	3.31 ± 0.32c	0.41 ± 0.04c	57.83 ± 5.47d

### Physiological responses of *A. cicer *roots under NaCl stress

**Fig. 1:**
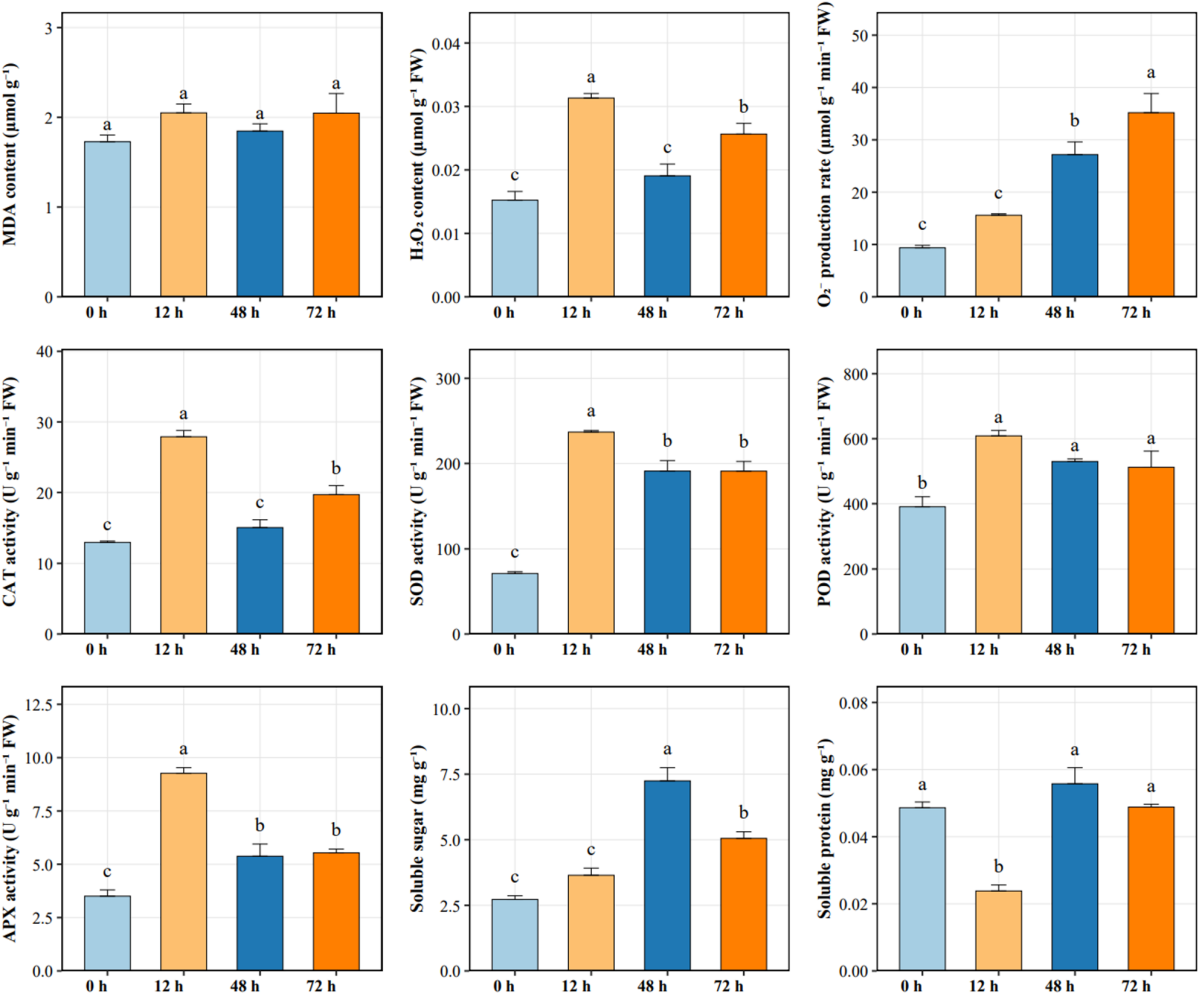
Physiological responses of *A. cicer* roots under NaCl stress at different time points (0 h, 12 h, 48 h, and 72 h). Different letters above bars indicate significant differences among time points according to Duncan’s multiple range test (*p* < 0.05). Data are presented as means ± SE (*n* = 3)

The physiological responses of *A. cicer* roots to NaCl stress indicated that both the superoxide anion production rate and malondialdehyde (MDA) content exhibited a continuous increase over the experimental period (Fig. 1). This suggests persistent oxidative damage and lipid peroxidation resulting from salt stress. In contrast, Catalase (CAT) activity and hydrogen peroxide (H_2_O_2_) content followed a trend of initial increase, subsequent decrease, and a second increase. Both the activity and content rose significantly at the early stage of stress, dropped at 48 h, and then increased again at 72 h. The activities of Superoxide dismutase (SOD), peroxidase (POD), ascorbate peroxidase (APX), and the soluble sugar content all showed a pattern of rising first, followed by a decline. The activities of these antioxidant enzymes and protective substances peaked at the initial time point, signifying the activation of the primary antioxidant defense mechanisms, before subsequently declining at 48 h and 72 h. Notably, the increase in APX activity was most pronounced at the 12 h mark. The pattern for soluble protein content was the reverse: it first decreased and then increased, reaching its maximum level at 48 h before decreasing again at 72 h.

### Metabolomic responses of *A. cicer* roots under NaCl stress

**Fig. 2:**
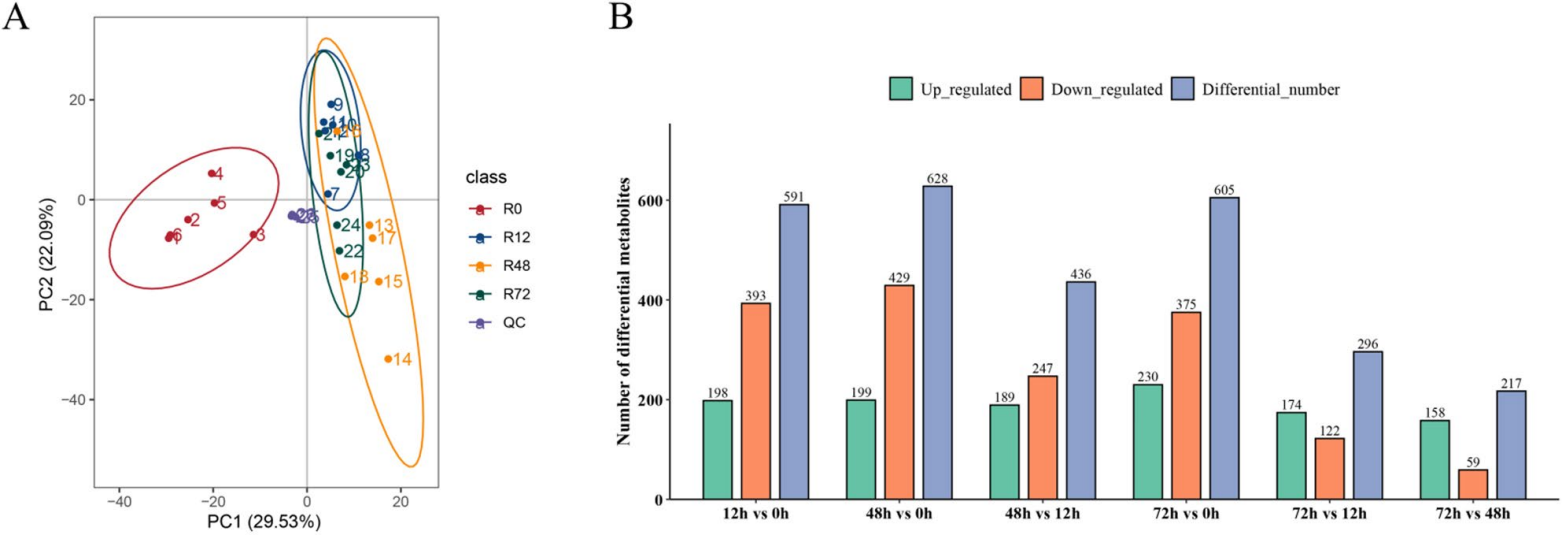
Principal Component Analysis and Differential Metabolite Statistic. **A** Principal component analysis (PCA) of metabolite profiles in *A. cicer* roots under NaCl stress at 0 h (R0), 12 h (R12), 48 h (R48), and 72 h (R72). **B** Number of differentially accumulated metabolites (DAMs) identified in pairwise comparisons between time points. Bars represent the number of upregulated (green), downregulated (orange), and total differential metabolites (blue) in each comparison group

Principal component analysis (PCA) of *A. cicer* root metabolite profiles under NaCl stress revealed clear separation among samples from different time points and tight clustering of replicates (Fig. 2A), indicating good reproducibility and overall data reliability. PLS-DA analysis confirmed the reliability of the model (Fig. S1). To evaluate the dynamic changes in metabolite expression under salt stress, pairwise comparisons of the four time points were conducted to identify differentially accumulated metabolites (DAMs) (Fig. 2B). The number of DAMs varied across comparisons. Among these, 12 h vs. 0 h and 48 h vs. 0 h had the highest number of upregulated metabolites, while 72 h vs. 0 h showed a large number of both upregulated and downregulated metabolites. Fewer DAMs were detected in later comparisons, such as 72 h vs. 12 h and 72 h vs. 48 h, suggesting a possible stabilization of metabolic responses at later stages.

### Identification and analysis of dams

**Fig. 3:**
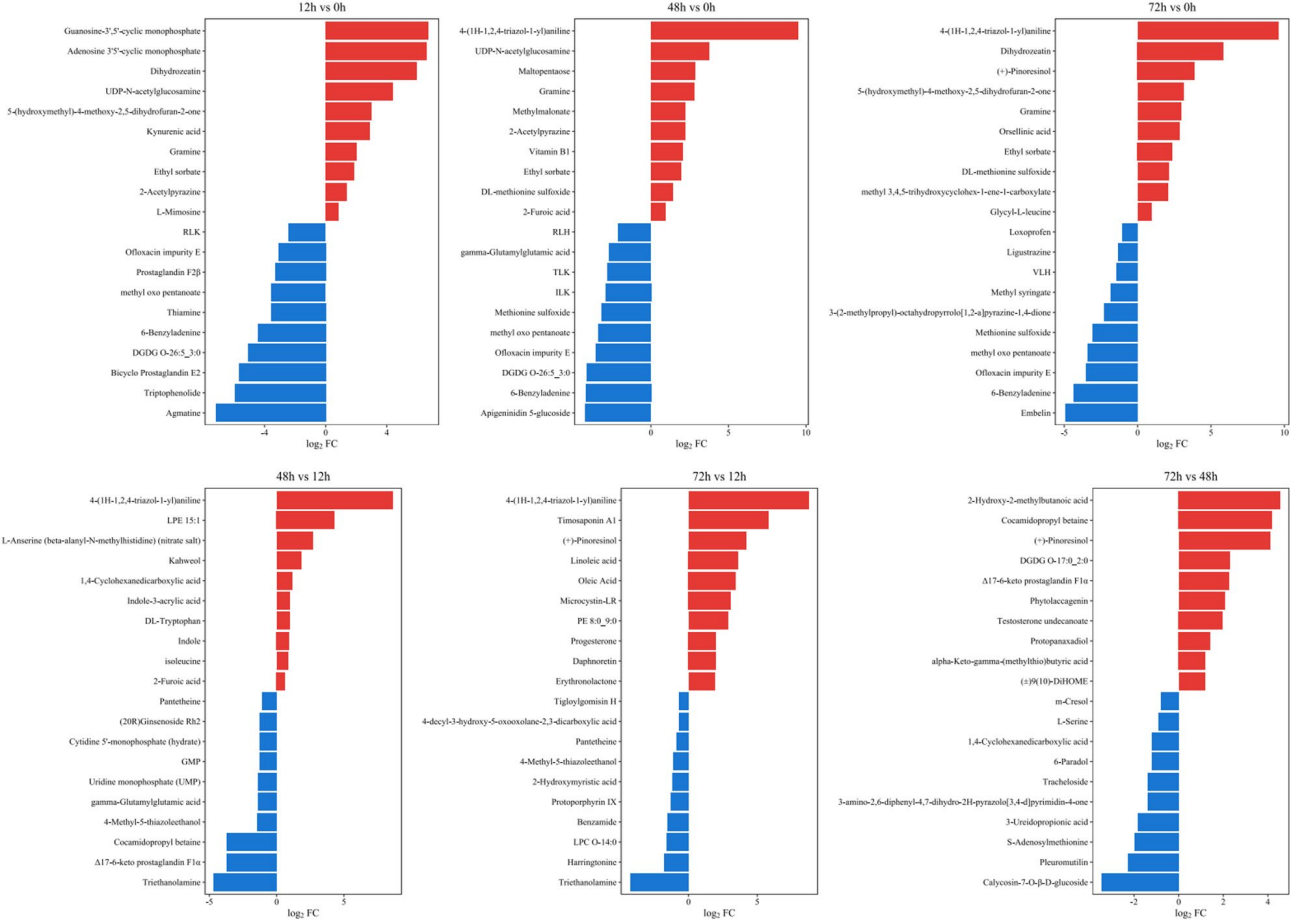
Top 10 upregulated (red) and downregulated (blue) differentially accumulated metabolites (DAMs) in *A. cicer* roots under NaCl stress at different time points

A comprehensive metabolomic analysis of A. cicer roots under NaCl stress identified a total of 1,677 metabolites. Based on stringent criteria (VIP > 1.0, fold change > 1.5 or < 0.667, and *p* < 0.05), distinct accumulation patterns of DAMs were observed across different time points (Fig. 3). Overall, downregulated metabolites outnumbered upregulated ones during the early and mid-term stages, suggesting a general suppression of metabolic activity under salt stress. At the early stage, a large number of DAMs were detected, with suppressed metabolites being nearly twice as abundant as accumulated ones. Notably, the most strongly accumulated metabolites included the signaling molecules guanosine-3′,5′-cyclic monophosphate and adenosine-3′,5′-cyclic monophosphate, as well as the cytokinin dihydrozeatin, indicating early activation of stress signaling and hormone-mediated regulatory pathways. In contrast, markedly suppressed metabolites such as agmatine and water-soluble vitamin E are associated with polyamine metabolism and antioxidant capacity, suggesting a transient inhibition of stress-protective metabolic processes. At the mid-term stage, the number of DAMs slightly increased, and accumulated metabolites were mainly related to signaling and hormonal regulation, including adenosine-3′,5′-cyclic monophosphate and dihydrozeatin. Meanwhile, the suppression of alkaloids and antioxidant-related compounds, such as pilocarpine, phellibaumin, and rosmarinic acid, reflects a reprogramming of secondary metabolism as roots adapt to prolonged NaCl stress.

### KEGG pathway enrichment of differentially accumulated metabolites in *A. cicer* roots under NaCl Stress

**Fig. 4:**
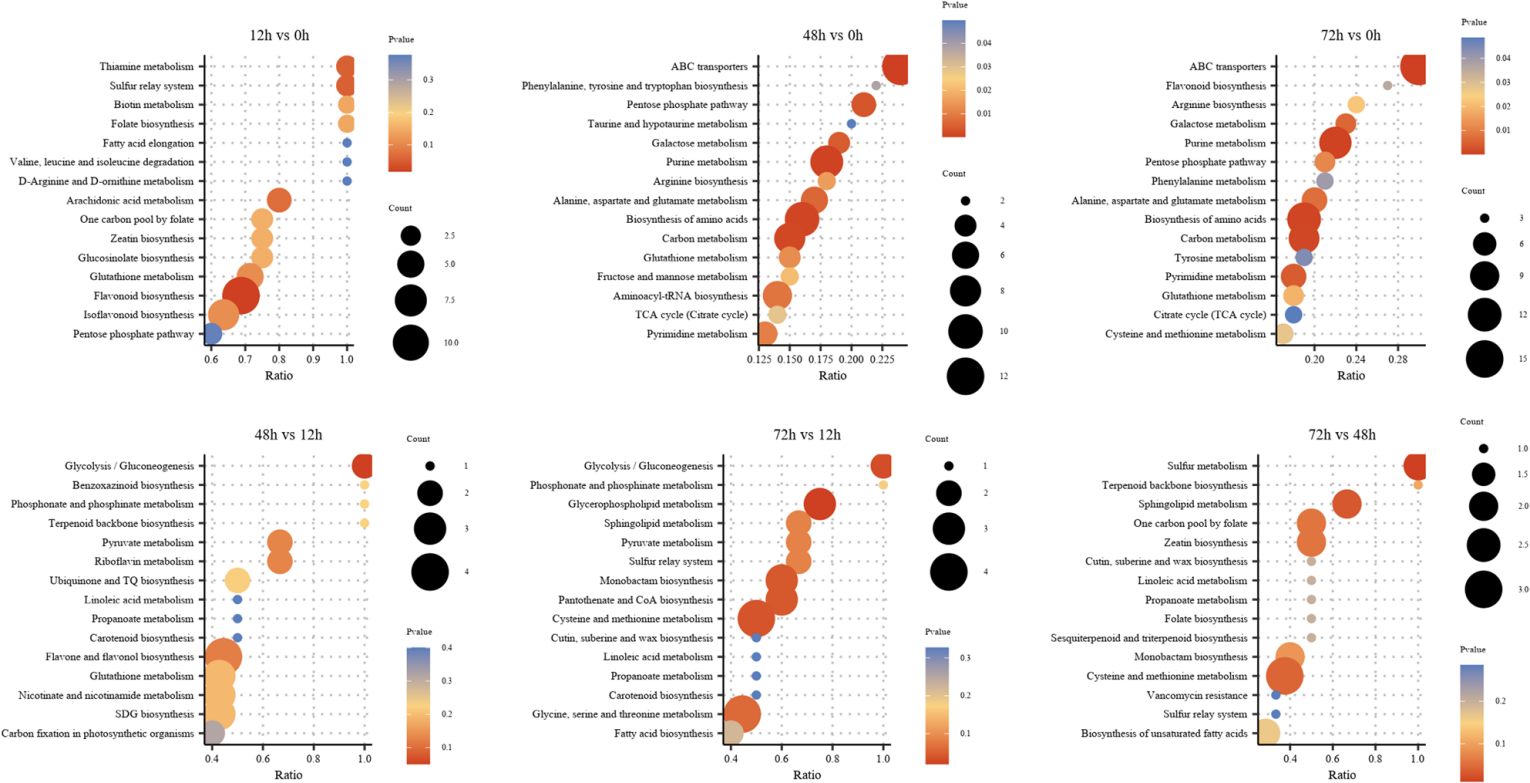
KEGG pathway enrichment analysis of differentially accumulated metabolites (DAMs) in *A. cicer* roots under NaCl stress. The top 20 enriched pathways for each comparison group are shown, based on metabolite count

To investigate the metabolic pathway responses of *A. cicer* roots under NaCl stress, KEGG enrichment analysis was performed on all identified differentially accumulated metabolites (DAMs) (Fig. 4). The results showed that metabolic responses varied dynamically across different time stages. In the early stage of stress, purine metabolism and cysteine and methionine metabolism were significantly enriched, indicating that roots rapidly respond to salt stress through signaling pathways and sulfur-containing amino acid–related pathways. Additionally, general metabolic pathways, ABC transporters, arachidonic acid metabolism, and pyrimidine metabolism were also enriched, reflecting active energy metabolism and stress responses during the early stage. In the mid-term stage, signaling and hormone-related pathways, amino acid metabolism, and certain secondary metabolic pathways remained enriched, suggesting that roots activate defense and homeostasis mechanisms to adapt to salt stress and lay the foundation for subsequent long-term adaptation. In the long-term stage, purine metabolism and sulfur-containing amino acid metabolism remained enriched, while core metabolic and certain secondary metabolic pathways were activated, indicating that roots maintain adaptive responses to prolonged salt stress through metabolic reprogramming.

### Clustering and expression pattern analysis of DAMs in *A. cicer* roots under NaCl stress

**Fig. 5:**
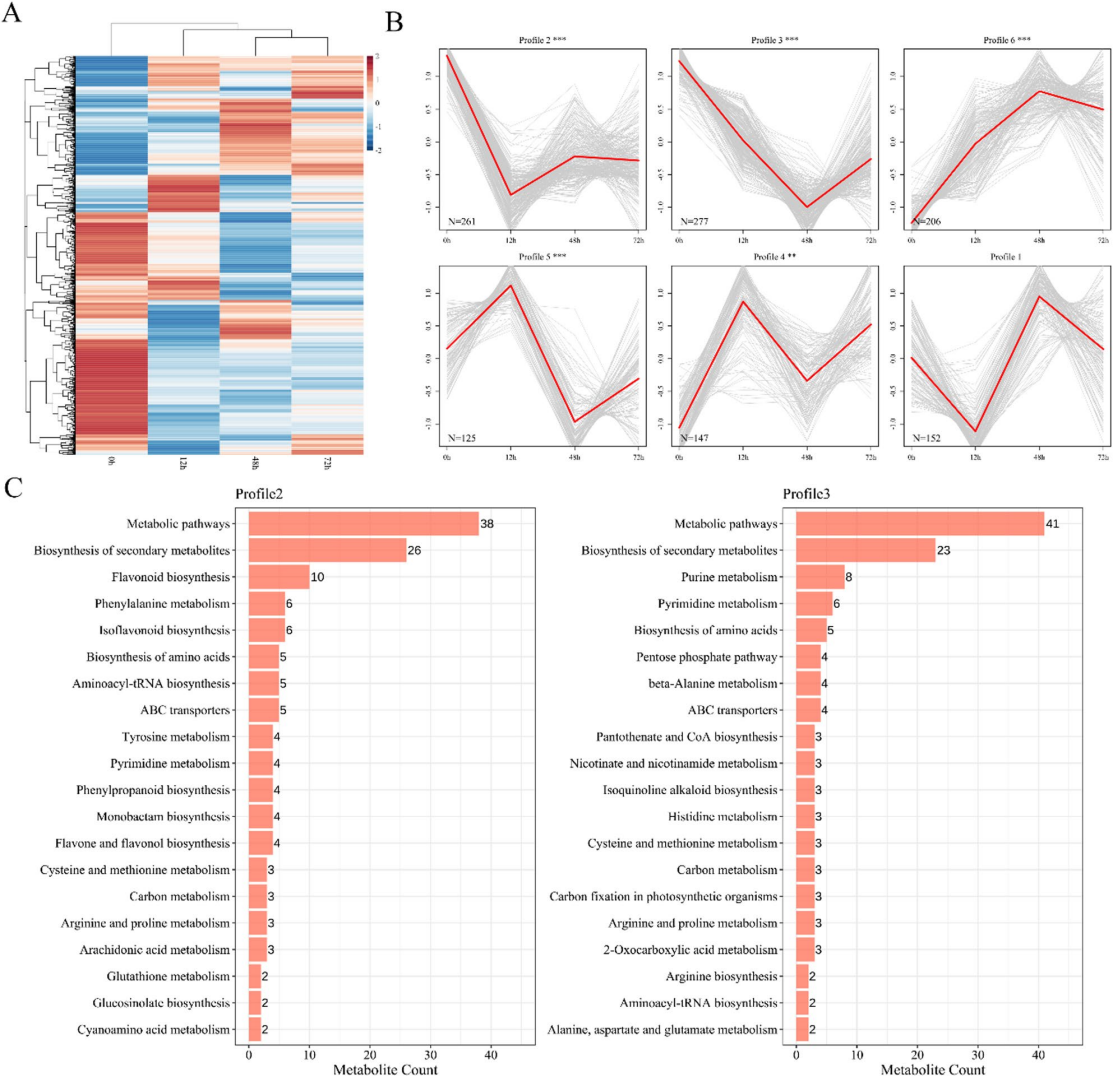
Clustering and Expression Pattern Analysis of DAMs. **A** Heatmap of differentially accumulated metabolites (DAMs) in *A. cicer* roots under NaCl stress across four time points (0 h, 12 h, 48 h, 72 h). **B** STEM clustering of DAMs into 6 temporal expression profiles. The red lines represent the model expression trend for each profile; grey lines represent individual metabolites. **C** KEGG pathway enrichment analysis of metabolites in Profile 2 (left) and Profile 3 (right). Bar charts indicate the number of metabolites enriched in each pathway

Hierarchical clustering of DAMs in *A. cicer* roots under NaCl stress revealed distinct expression patterns among the treatment groups (Fig. 5A). Temporal analysis using STEM grouped DAMs into six expression profiles, of which five showed significant enrichment, highlighting dominant dynamic trends in response to salt stress (Fig. 5B). KEGG enrichment of these significant profiles indicated activation of multiple metabolic and biosynthetic pathways, including secondary metabolite biosynthesis, flavonoid and phenylpropanoid metabolism, and nucleotide and amino acid biosynthesis (Fig. 5C). These results demonstrate a coordinated and time-specific metabolic response of *A. cicer* roots to prolonged NaCl stress.

## Discussion

Soil salinization is a major abiotic stress limiting plant growth and crop yield [26]. In this study, we investigated the metabolic response of *A. cicer* roots under NaCl stress by metabolomic analysis at 0 h, 12 h (early), 48 h (mid-term), and 72 h (late) after treatment. Physiological observations revealed that low salt concentration (50 mM) promoted seed germination and seedling growth, whereas high salt concentration (150 mM) suppressed growth and sharply reduced the root-to-shoot ratio, suggesting severe osmotic stress and ion toxicity. Under high-salinity conditions, metabolomic profiling captured dynamic metabolic adjustments associated with stress adaptation.

High salt stress led to continuous oxidative pressure, as reflected by the accumulation of MDA and superoxide anions. In response, *A. cicer* rapidly activated its antioxidant defense system, including SOD, POD, and APX, with activities peaking during the early phase. The subsequent decline in enzyme activity, together with the persistent accumulation of ROS in later stages, indicates that prolonged salt exposure can challenge the balance of the antioxidant system [27, 28]. These physiological dynamics are closely mirrored by metabolomic adjustments: KEGG enrichment analysis highlighted purine metabolism and cysteine/methionine metabolism as core pathways consistently enriched across all time points (Fig. 4), supporting energy supply, redox homeostasis, and sulfur-based antioxidant defense. Additional pathways, including phenylpropanoid biosynthesis, pyrimidine metabolism, and ABC transporters, were also significantly enriched, collectively forming a metabolic framework for salt stress adaptation. Purine metabolism ensures the supply of ATP and nucleotide-derived signaling molecules [29, 30], while cysteine/methionine metabolism provides sulfur and nitrogen skeletons for the synthesis of non-enzymatic antioxidants such as GSH, consistent with the observed oxidative stress conditions [31, 32].

During the early response phase, cGMP and cAMP accumulated rapidly, consistent with engagement of cyclic nucleotide–associated signaling processes that regulate ion channels and redox homeostasis [33, 34]. Simultaneously, S-adenosyl-L-methionine (SAM), a precursor for polyamine biosynthesis, exhibited dynamic changes, indicating potential involvement of polyamine-related networks in early stress adaptation. Polyamines can modulate H₂O₂ levels and membrane transport, contributing to redox regulation and ion homeostasis [35, 36]. Together, the coordinated changes in cyclic nucleotides and SAM indicate an integrated metabolic response that supports cellular homeostasis under early salt stress.

Beyond these core pathways, *A. cicer* employed additional adaptive strategies. Soluble sugars peaked during the mid-term phase, reflecting osmolyte accumulation for maintaining cell turgor and alleviating osmotic stress [37, 38]. Secondary metabolite pathways, especially flavonoid biosynthesis, were also enriched, enhancing antioxidant capacity in response to sustained ROS accumulation [39]. The accumulation of hormones such as dihydrozeatin further suggests a role for hormonal regulation in maintaining root growth and coordinating stress responses [40]. Concurrent downregulation of primary metabolites such as phenylalanine and L-methionine reflects a metabolic shift from growth-related processes toward defense and stress adaptation [41, 42].

Collectively, these results indicate that *A. cicer* tolerance to salt stress relies on a dynamically coordinated network encompassing energy metabolism, redox regulation, osmotic adjustment, and hormone-associated responses. Early cAMP/cGMP accumulation coincides with the onset of oxidative and ionic stress, whereas sustained increases in soluble sugars and secondary metabolites support osmotic balance and antioxidant protection. Repeated enrichment of purine, cysteine/methionine, phenylpropanoid, and flavonoid pathways highlights their central roles in energy provision, sulfur-based antioxidant defense, and secondary metabolite–mediated protection, providing an integrated metabolic framework for salt adaptation in *A. cicer*.

## Conclusion

This study utilized metabolomic analysis to systematically investigate the adaptive response of *A. cicer* roots to NaCl stress over a 72 h period. The findings revealed a crucial biphasic growth response, where low salinity (50 mM) promoted early development, while high salinity (150 mM) induced severe oxidative stress and growth suppression. Metabolomic profiling confirmed that the adaptive strategy is underpinned by extensive metabolic adjustment involving key secondary messengers and structural compounds. Differentially accumulated metabolites were consistently enriched in strategic metabolic pathways, including purine metabolism, cysteine and methionine metabolism, phenylpropanoid biosynthesis, flavonoid biosynthesis, glutathione metabolism, and arginine and proline metabolism. Notably, the early and sustained upregulation of signaling molecules (cAMP, cGMP) and protective compounds (dihydrozeatin, soluble sugars) highlights their roles in initiating defense mechanisms, maintaining cellular homeostasis, and enabling osmotic adjustment. These findings provide a comprehensive metabolic basis for understanding the molecular mechanisms of salt tolerance in *A. cicer* and offer potential targets for the development of salt-tolerant varieties.

## Supplementary Information

Supplementary Material 1.

## Data Availability

The raw metabolomics data generated in this study are available from the corresponding author upon reasonable request. All other analytical data are available within the article.

## Abbreviations

APX: Ascorbate peroxidase
ATP: Adenosine triphosphate
cAMP: Cyclic adenosine monophosphate
cGMP: Cyclic guanosine monophosphate
CAT: Catalase
DAMs: Differentially accumulated metabolites
GSH: Glutathione
KEGG: Kyoto Encyclopedia of Genes and Genomes
NBT: Nitrobluetetrazolium
POD: Peroxidase
ROS: Reactive oxygen species
SOD: Superoxide dismutase
SPSS: Statistical Package for the Social Sciences

## References

[1] Liang X, Li J, Yang Y, Jiang C, Guo Y. Designing salt stress-resilient crops: current progress and future challenges. J Integr Plant Biol. 2024;66(3):303–29. 38108117 10.1111/jipb.13599

[2] Zarei M, Shabala S, Zeng F, Chen X, Zhang S, Azizi M, Rahemi M, Davarpanah S, Yu M, Shabala L. Comparing kinetics of xylem ion loading and its regulation in halophytes and glycophytes. Plant Cell Physiol. 2020;61(2):403–15. 31693150 10.1093/pcp/pcz205

[3] Sui N, Wang Y, Liu S, Yang Z, Wang F, Wan S. Transcriptomic and physiological evidence for the relationship between unsaturated fatty acid and salt stress in peanut. Front Plant Sci. 2018;9:7. 10.3389/fpls.2018.00007 PMC5786550 29403517

[4] Khan N, Bano A, Rahman MA, Rathinasabapathi B, Babar MA. UPLC-HRMS-based untargeted metabolic profiling reveals changes in Chickpea (*Cicer arietinum*) metabolome following long-term drought stress. Plant Cell Environ. 2019;42(1):115–32. 29532945 10.1111/pce.13195 PMC7379973

[5] Deinlein U, Stephan AB, Horie T, Luo W, Xu G, Schroeder JI. Plant salt-tolerance mechanisms. Trends Plant Sci. 2014;19(6):371–9. 24630845 10.1016/j.tplants.2014.02.001 PMC4041829

[6] Cabot C, Sibole JV, Barceló J, Poschenrieder C. Lessons from crop plants struggling with salinity. Plant Sci. 2014;226:2–13. 25113445 10.1016/j.plantsci.2014.04.013

[7] Chen M, Yang Z, Liu J, Zhu T, Wei X, Fan H, et al. Adaptation mechanism of salt excluders under saline conditions and its applications. Int J Mol Sci. 2018. 10.3390/ijms19113668. 30463331 10.3390/ijms19113668 PMC6274768

[8] Baxter I, Hosmani PS, Rus A, Lahner B, Borevitz JO, Muthukumar B, et al. Root suberin forms an extracellular barrier that affects water relations and mineral nutrition in *Arabidopsis*. PLoS Genet. 2009;5(5):e1000492. 19461889 10.1371/journal.pgen.1000492 PMC2679201

[9] Ho WWH, Hill CB, Doblin MS, Shelden MC, van de Meene A, Rupasinghe T, et al. Integrative multi-omics analyses of Barley rootzones under salinity stress reveal two distinctive salt tolerance mechanisms. Plant Commun. 2020;1(3):100031. 33367236 10.1016/j.xplc.2020.100031 PMC7748018

[10] Jiao Y, Bai Z, Xu J, Zhao M, Khan Y, Hu Y, et al. Metabolomics and its physiological regulation process reveal the salt-tolerant mechanism in *Glycine soja* seedling roots. Plant Physiol Biochem. 2018;126:187–96. 29525442 10.1016/j.plaphy.2018.03.002

[11] Dersch LM, Beckers V, Wittmann C. Green pathways: metabolic network analysis of plant systems. Metab Eng. 2016;34:1–24. 26704307 10.1016/j.ymben.2015.12.001

[12] Lee SJ, Jeong EM, Ki AY, Oh KS, Kwon J, Jeong JH, et al. Oxidative defense metabolites induced by salinity stress in roots of *Salicornia herbacea*. J Plant Physiol. 2016;206:133–42. 27770750 10.1016/j.jplph.2016.08.015

[13] Li H, Tang X, Yang X, Zhang H. Comprehensive transcriptome and metabolome profiling reveal metabolic mechanisms of *Nitraria sibirica* Pall. to salt stress. Sci Rep. 2021;11(1):12878. 34145354 10.1038/s41598-021-92317-6 PMC8213879

[14] Kim JK, Bamba T, Harada K, Fukusaki E, Kobayashi A. Time-course metabolic profiling in *Arabidopsis thaliana* cell cultures after salt stress treatment. J Exp Bot. 2007;58(3):415–24. 17118972 10.1093/jxb/erl216

[15] Rajkumari N, Chowrasia S, Nishad J, Ganie SA, Mondal TK. Metabolomics-mediated elucidation of rice responses to salt stress. Planta. 2023;258(6):111. 37919614 10.1007/s00425-023-04258-1

[16] Joshi R, Sahoo KK, Singh AK, Anwar K, Pundir P, Gautam RK, et al. Enhancing trehalose biosynthesis improves yield potential in marker-free transgenic rice under drought, saline, and sodic conditions. J Exp Bot. 2020;71(2):653–68. 31626290 10.1093/jxb/erz462 PMC6946002

[17] Chun HJ, Baek D, Cho HM, Jung HS, Jeong MS, Jung WH, et al. Metabolic adjustment of *Arabidopsis* root suspension cells during adaptation to salt stress and mitotic stress memory. Plant Cell Physiol. 2019;60(3):612–25. 30496500 10.1093/pcp/pcy231

[18] Pitcher LR, MacAdam JW, Ward RE, Han KJ, Griggs TC, Dai X. Beef steer performance on irrigated monoculture legume pastures compared with grass- and concentrate-fed steers. Animals. 2022. 10.3390/ani12081017. 35454263 10.3390/ani12081017 PMC9032127

[19] Issah G, Schoenau JJ, Lardner HA, Knight JD. Nitrogen fixation and resource partitioning in alfalfa (*Medicago sativa* L.), cicer milkvetch (*Astragalus cicer* L.) and sainfoin (*Onobrychis viciifolia Scop*.) using 15N enrichment under controlled environment conditions. Agronomy. 2020. 10.3390/agronomy10091438.

[20] Zhang JY, Zhang LJ, Zhao QS, Xia M. Salt tolerance of several leguminous forage grasses during seed germination. Chin J Grassland. 2012;34(4):116–20.

[21] Zhang Y, Li D, Dirk LMA, Downie AB, Zhao T. *ZmAGA1* hydrolyzes RFOs late during the lag phase of seed germination, shifting sugar metabolism toward seed germination over seed aging tolerance. J Agric Food Chem. 2021;69(39):11606–15. 34553917 10.1021/acs.jafc.1c03677

[22] Kaur H, Bhatla SC. Melatonin–nitric oxide interaction modulates catalase activity and hydrogen peroxide homeostasis in sunflower seedling cotyledons accompanying NaCl stress. J Plant Growth Regul. 2023;42(10):6261–72.

[23] Nazir F, Hussain A, Fariduddin Q. Hydrogen peroxide modulate photosynthesis and antioxidant systems in tomato (*Solanum lycopersicum* L.) plants under copper stress. Chemosphere. 2019;230:544–58. 31125883 10.1016/j.chemosphere.2019.05.001

[24] Sardar H, Khalid Z, Ahsan M, Naz S, Nawaz A, Ahmad R, et al. Enhancement of salinity stress tolerance in lettuce (*Lactuca sativa* L.) via foliar application of nitric oxide. Plants. 2023. 10.3390/plants12051115. 36903975 10.3390/plants12051115 PMC10005404

[25] Fan X, Bao G, Xie Y, Jiang Y, Fan C, Li G. Seedlings of rye (*Secale cereale*) respond to freeze-thaw, alkaline salt, and *Solanum rostratum* Dunal extract combined stress by increasing soluble protein and antioxidant enzyme activity. Funct Plant Biol. 2025. 10.1071/FP24229. 40245264 10.1071/FP24229

[26] Fu J, Liu Y, Liu X, Guo M, Gao J, Yang M, et al. Screening of saline-alkali tolerant microorganisms and their promoting effects on rice growth under saline-alkali stress. J Clean Prod. 2024;481:144176.

[27] Zulfiqar F, Ashraf M. Antioxidants as modulators of arsenic-induced oxidative stress tolerance in plants: an overview. J Hazard Mater. 2022;427:127891. 34848065 10.1016/j.jhazmat.2021.127891

[28] Goyal V, Jhanghel D, Mehrotra S. Emerging warriors against salinity in plants: nitric oxide and hydrogen sulphide. Physiol Plant. 2021;171(4):896–908. 33665834 10.1111/ppl.13380

[29] Liu L, Zhang P, Feng G, Hou W, Liu T, Gai Z, et al. Salt priming induces low-temperature tolerance in sugar beet via xanthine metabolism. Plant Physiol Biochem. 2023;201:107810. 37321038 10.1016/j.plaphy.2023.107810

[30] Han M, Cui R, Wang D, Huang H, Rui C, Malik WA, et al. Combined transcriptomic and metabolomic analyses elucidate key salt-responsive biomarkers to regulate salt tolerance in cotton. BMC Plant Biol. 2023;23(1):245. 37161359 10.1186/s12870-023-04258-z PMC10170727

[31] Liu Y, Zheng J, Ge L, Tang H, Hu J, Li X, et al. Integrated metabolomic and transcriptomic analyses reveal the roles of alanine, aspartate and glutamate metabolism and glutathione metabolism in response to salt stress in tomato. Sci Hortic. 2024;328:112911.

[32] Patel M, Parida AK. Salinity alleviates arsenic stress-induced oxidative damage via antioxidative defense and metabolic adjustment in the root of the halophyte *Salvadora persica*. Planta. 2023;258(6):109. 37907764 10.1007/s00425-023-04263-4

[33] Zhao Y, Liu Y, Ji X, Sun J, Lv S, Yang H, et al. Physiological and proteomic analyses reveal cAMP-regulated key factors in maize root tolerance to heat stress. Food Energy Secur. 2021;10(4):e309.

[34] Wang P, Nie X, Ye Y, Abid MA, Liang C, Zhang R, et al. Forskolin improves salt tolerance of *Gossypium hirsutum* L. by upregulation of *GhLTI65*. Ind Crops Prod. 2023;201:116900.

[35] Zhang X, Bao Z, Gong B, Shi Q. S-adenosylmethionine synthetase 1 confers drought and salt tolerance in transgenic tomato. Environ Exp Bot. 2020;179:104226.

[36] Alhammad BA, Seleiman MF, Harrison MT. Hydrogen peroxide mitigates Cu stress in wheat. Agriculture. 2023. 10.3390/agriculture13040862.

[37] Nawaz M, Hassan MU, Chattha MU, Mahmood A, Shah AN, Hashem M, et al. Trehalose: a promising osmo-protectant against salinity stress—physiological and molecular mechanisms and future prospective. Mol Biol Rep. 2022;49(12):11255–71. 35802276 10.1007/s11033-022-07681-x

[38] Sánchez P, Castro-Cegrí A, Sierra S, Garrido D, Llamas I, Sampedro I, et al. The synergy of halotolerant PGPB and mauran mitigates salt stress in tomato (*Solanum lycopersicum*) via osmoprotectants accumulation. Physiol Plant. 2023;175(6):e14111. 38148230 10.1111/ppl.14111

[39] Baozhu L, Ruonan F, Yanting F, Runan L, Hui Z, Tingting C, et al. The flavonoid biosynthesis regulator PFG3 confers drought stress tolerance in plants by promoting flavonoid accumulation. Environ Exp Bot. 2022;196:104792.

[40] Long J, Liu D, Qiao W, Wang Y, Miao Y, Baosai H. Response of *Elymus nutans* Griseb. seedling physiology and endogenous hormones to drought and salt stress. Sci Rep. 2024;14(1):17810. 39090163 10.1038/s41598-024-68894-7 PMC11294584

[41] Li X, Liu Y, Hu W, Yin B, Liang B, Li Z, et al. Integrative physiological, metabolomic, and transcriptomic analysis reveals the drought responses of two apple rootstock cultivars. BMC Plant Biol. 2024;24(1):219. 38532379 10.1186/s12870-024-04902-2 PMC10964572

[42] Simpson JP, Olson J, Dilkes B, Chapple C. Identification of the Tyrosine- and Phenylalanine-Derived soluble metabolomes of sorghum. Front Plant Sci. 2021;12:714164. 10.3389/fpls.2021.714164 PMC8476951 34594350

