# Supplementary material for: Metabolomic insights into the mechanisms of chickpea milkvetch (Astragalus Cicer L.) response to salt stress
Source: BMC Plant Biol. 2026 Feb 26;26:599. doi: 10.1186/s12870-026-08375-3 (PMC13041155; doi:10.1186/s12870-026-08375-3)
Supplement: Supplementary file 1 — Supplementary Material 1. [file 12870_2026_8375_MOESM1_ESM.docx]

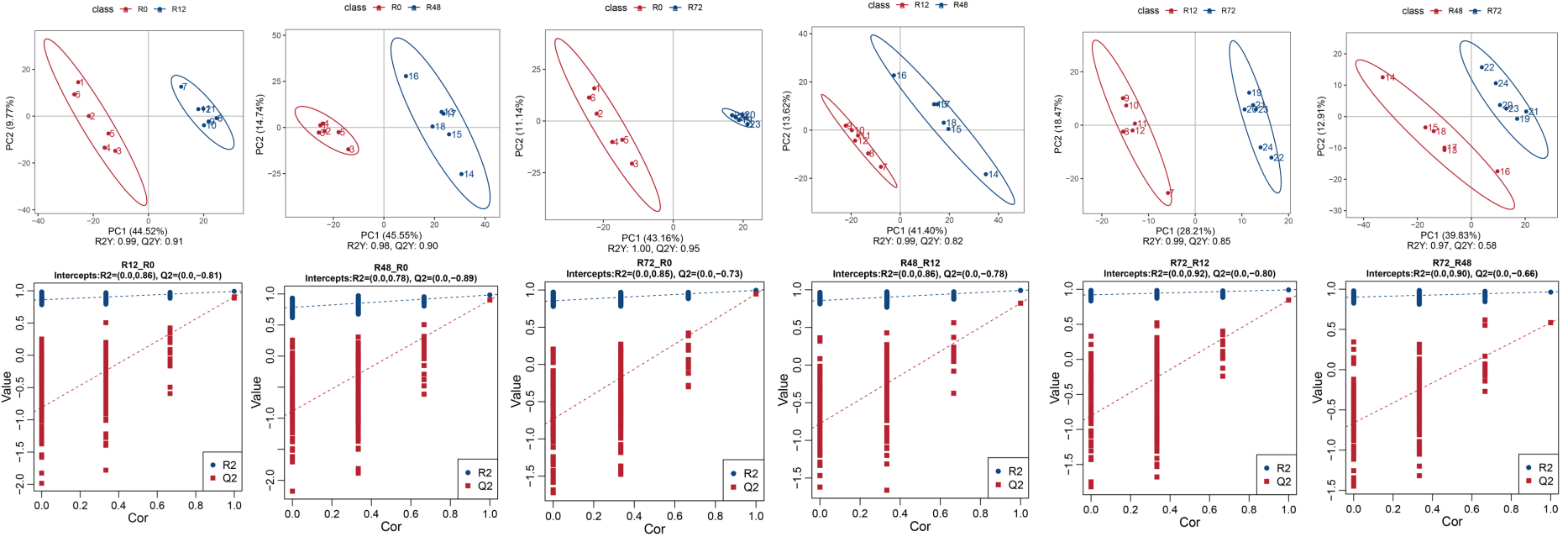


Figure S1. PLS-DA analysis of root metabolomic profiles of *Astragalus cicer* under different treatment times.
